# Anaesthesiologist-provided pre-hospital advanced airway management in children

**DOI:** 10.1186/1757-7241-23-S2-A22

**Published:** 2015-09-11

**Authors:** M Tarpgaard, TM Hansen, L Rognås

**Affiliations:** 1 Department of Pre-hospital Medical Services, Central Denmark Region, Denmark

## Background

Pre-hospital advanced airway management has been named one of the top-five research priorities in physician-provided pre-hospital critical care [1]. Few studies have been made on paediatric pre-hospital advanced airway management. The aim of this study was to investigate first-pass success rates and complications related to pre-hospital advanced airway management in patients younger than 16 years of age treated by pre-hospital critical care teams in the Central Denmark Region (1.3 million inhabitants).

## Method

A prospective descriptive study based on data collected from eight anaesthesiologist-staffed pre-hospital critical care teams between February 1st 2011 and November 1st 2012.

## Results

Of a total of 25 000 pre-hospital critical care missions, the pre-hospital critical care anaesthesiologists attempted endotracheal intubation in 25 children, 13 of which were less than 2 years old.

In one patient, a neonate (600g birth weight), endotracheal intubation failed. The patient was managed by uneventful bag-mask ventilation.

All other children had their tracheas successfully intubated by the pre-hospital critical care anaesthesiologists.

Over-all first pass success-rate was 75.0 %. In the group of patients younger than 2 years old, first pass success-rate was 53.8 %.

The overall rate of airway management related complications was 20 % in children younger than 16 years of age and 38 % in children younger than 2 years of age (n=13). No deaths, cardiac arrests or severe bradycardia (heart rate <60) occurred in relation to pre-hospital advanced airway management.

## Discussion

Compared to the adult population [2] the overall first-pass success rate is low. The complication rates (hypoxia, hypotension, aspiration and oesophageal intubations) in the paediatric population are higher than previously described in our pre-hospital advanced airway management patient population as a whole [2]. This illustrates that young children may represent a substantial pre-hospital airway management challenge even for experienced pre-hospital critical care anaesthesiologists.
